# Collectanea Medica, Consisting of Anecdotes, Facts, Extracts; Illustrations, Queries, Suggestions, &c.

**Published:** 1818-10

**Authors:** 


### COLLECTANEA MEDICA,

CONSISTING OF

### ANECDOTES, FACTS, EXTRACTS; ILLUSTRATIONS, QUERIES, SUGGESTIONS, &c.

RELATING TO THE

*History or the Art of Medicine, and the Auxiliary Sciences.*

Quicquid agunt medici, nostri farrago libelli.

***On Inedia.*** **(From Mr. Good's Physiological Nosology.)**

As gluttony, or a desire to be perpetually eating, may be acquired by habit, so may fasting. (See *Darwin, Esuries* 1, 2, 3.) In this metropolis the idle always eat more frequent meals than the busy. To what extent progressive habit might enable man to protract fasting without inconvenience or disease, has never, perhaps, been fairly tried: but the appetite of hunger seems, from various cases, almost as capable of being triumphed over as other appetites, and the body of being nourished by a very trifling quantity of food, and for many weeks, perhaps months, even by water alone. (See *Marcardier, in Journal de Medicine,* tom. xxxiii. *Schenck,* lib. 3, obs. 39. *Waldschmid, Dissertatio de his qui diu vivunt sine alimento, kil.* 1711.

One of the best known and best marked examples in our own day, is that of Ann Moore, of Tutbury. This woman has, indeed, been sufficiently ascertained to be a gross impostor, in pretending to be able to live *without any food whatever;* but she seems, from long habit, to have lost all pleasurable desire for food, and to be capable of subsisting upon very simple liquids alone. She was at first induced to this habit by an extreme difficulty of deglutition, and she at length carried the habit so far, as, by deception, easily to excite a general belief that she never swallowed any thing, either liquid or solid. The intelligent committee, who so laudably formed themselves into a watch to determine the state of the fact, by a constant attendance upon her person for a month, sufficiently proved that she could not live for ten days without swallowing some portion of liquid. In their report they tell us, that "on the eighth day she was exceedingly distressed," her pulse had increased until it amounted to one hundred and forty-five strokes in a minute; and "so far was she reduced on the ninth day, that she became in danger of expiring;" while, a few hours afterwards, when she was compelled to confess the imposture she had practised, "the pulse at one wrist had nearly ceased, and the other seemed drawn to a thread." Yet, "on the whole," say the committee, "though this woman is a base impostor with respect to her pretence of *total* abstinence from all food whatever, liquid or solid, yet she *can, perhaps, endure the privation of solid food longer than any other person.* It is thought by those best acquainted with her, that she existed on a mere trifle, and from hence came the temptation to say that she did not take any thing. The daughter says, that her mother's principal food is tea, and there is reason to believe this to be true."—*Full Exposure of Ann Moore, the pretended Fasting Woman of Tutbury.*

Hildanus and Haller have collected numerous instances of *complete fasting* for very long periods of time, in some instances for not less than sixteen years, but most of them are too loosely recorded to be relied upon. There are other cases of this kind, however, that seem entitled to more attention; (See *Willan's Medical Commun.* ii. 113.) where life was supported sixty days on water with a little orange juice. Dr. Eccles relates a case in the Edinburgh Medical Essays for 1720, of a young lady, about sixteen years of age, who, in consequence of the sudden death of an indulgent father, was thrown into a state of tetanus or rigidity of all the muscles of the body, and especially those of deglutition, accompanied with a total loss of desire for food, as well as incapacity of swallowing it, for two long and distinct periods of time: in the first instance for thirty-four, and in the second, which occurred shortly afterwards, for fifty-four days; "all which time (observes the writer) of her first and second fastings, she declared she had no sense of hunger and thirst; and when they were over, she had not lost much of her flesh." Sauvages alludes to a similar effect produced by religious mania, and nymphomania. *Nosol.* ii. p. 805. *Asthenia abstinentium.* A case is related in the Philosophical Transactions for the year 1681, of Margaret Lower, who, for four months, was ordered to desist from food of every kind, solid as well as liquid, in consequence of the whole she took, and even the glysters given her, being instantly vomited up.

Four men were preserved in a mine, from which, in consequence of an accident, they were incapable of being extricated for twenty-four days, without other food than water. *Phil. Trans.* 1684.—A man, said to have lived eighteen years on water, with occasionally a little clarified whey, and locked up for twenty days in close confinement, with water alone, to prove whether there were any imposition: meagre, and supposed to have no evacuations, but in good health, and pursued husbandry. *Id.* 1742.—A woman, from epileptic fits, when a girl of fifteen, took to her bed, lost her appetite, and was attacked with locked-jaw, which, with a few short intervals, continued for four years: was, on two or three occasions, induced to take a little water, and her mouth was at times moistened with wetted linen through a cavity in her teeth; but swallowed nothing else. After this period, began gradually to recover from the tetanus, but had no desire for food; and, twelve years from the attack, when able to walk upright, took no more food than sufficient for an infant of two years of age. Had no egesta, but when ingesta, which were proportioned to each other; but sometimes a dewy softness on her skin. *Dr. Mackenzie, Phil. Trans.* vol. lxvii. 1777. This case is authenticated by numerous witnesses of high respectability, and is entitled to peculiar attention. Case of a woman, who lost all desire of taking food by a fall from her horse into water during her first menstruation, at the age of eighteen: for fifty years scarcely ever took solids, her chief food being whey in the summer, and milk, milk and water, or pure water, in the winter: had frequent retchings, which were cured by smoking tobacco: for the space of sixteen years had only one stool annually; menstruation never recurred, but occasional vomitings of blood. *Edin. Med. Ess.* vol. vi. p. 6. 4th edit.

---

### *Curious Fact respecting Respiration.*

There are no actions of the human body which appear more completely associated with each other than those of the intercostal muscles, on the different sides of the thorax. The simultaneous movements of the different ribs, and the regular dilatation of the thorax, seem to depend on this association. The following curious fact, related in the Transactions of the Philosophical Society of Philadelphia, (vol. i. new series) by Dr. Wistar, will, however, evince that the action of the intercostal muscles, of one side of the thorax, may be completely suspended, while the muscles of the other side perform their accustomed motions most perfectly. It also affords a satisfactory explanation of this unusual occurrence.

A gentleman was attended by Dr. Wistar and Dr. Monges, for an hæmoptysis, which occasioned his death. During his indisposition, they observed that one side of his thorax was neither dilated nor contracted during respiration, and that the ribs on that side were perfectly quiescent, although those of the other side performed more motion than usual, and, therefore, dilated that side of the thorax to an uncommon degree. They first noticed this some days before his death, and it continued so without alteration during the remainder of his life. He did not complain of pain in the side which was without motion, but said he had sensations on the other side, which he believed were produced by the passage of blood from the ruptured blood vessels. Some years before, he had suffered with hæmoptysis, and a consequent cough and expectoration; but he recovered from this so much, that he was strong and rather corpulent at the time of his last attack. Upon dissection, the cause of this extraordinary mode of respiration was sufficiently obvious: that cavity of the thorax which was without motion was filled with pus; the volume of the lung of that side was greatly diminished, and the cellular structure of the organ entirely abolished.

---

In the Cottonian Manuscripts at the British Museum, *Lib. Vitell.* c. 3, is the following prescription for a *specific for gout;* or, as it is there called, *fot-adl:*—"Take the herb *datulus ortitulosa,* which we call greater cranleac (a species of iris, or flag-flower;) take the heads of it and dry them very much, and take thereof a penny-weight and a half; and the pear-tree and Roman bark, and cummin, and a fourth part of laurel berries; and, of the other herbs, half a penny-weight of each, and six pepper-corns; and grind all to dust, and put two egg-shells full of wine.—This is true leech-craft. Give it to the man till he be well."

---

*Hints relative to the Nature and Origin of the Plague, and the Mode in which it is disseminated.* From Mr. Jackson's Account of the Empire of Morocco.

"The symptoms of this plague varied in different patients: the variety of age and constitution gave it a like variety of appearance and character. In some it manifested itself by a sudden and violent shivering; in others, by a sudden delirium, succeeded by great and unquenchable thirst. Cold water was eagerly resorted to by the unwary and imprudent, and proved fatal to those who indulged in its momentary relief. Some had one, two, or more buboes, which formed themselves and became often as large as a walnut in the course of a day; others had a similar number of carbuncles. Those who were affected with a shivering, having no bubo, carbuncle, spots, or any other disfiguration, were invariably carried off in less than twenty-four hours; and the body of the deceased became quickly putrified, so that it was indispensably necessary to bury it in a few hours after dissolution. The European merchants shut themselves up in their respective houses, as is the practice in the Levant. I did not take this precaution, but occasionally rode out to take exercise on horseback. My daily observations convinced me that the epidemy was not caught by approach, unless that approach was accompanied by our inhaling of the breath, or by touching the infected person."

He afterwards states, that "families which had retired to the country to avoid the infection, on returning to town, when all infection had apparently ceased, were generally attacked and died." "After the mortality had subsided at Mogodore, a corps of troops arrived from the city of Terodant, in the province of Luse, where the plague had been raging and had subsided: these troops, after remaining three days at Mogodore, were attacked with the disease, and it raged *exclusively* among them for about a month, though they were not confined to any particular quarter, many of them having had apartments in the houses of the inhabitants of the town."

---

### *Lunatic Hospital at Avignon.*

The following account of the above institution, which has been officially made public, contains some remarks on the physical and moral management of insane patients, so important, particularly as being the result of experience, that we have considered it worthy a place in the department of our Journal we devote to subjects particularly calculated to excite reflection in our readers.

The hospital is under the management of the Lady Supérior, and twenty-five sisters of the *Soeurs de la Charité;* a director, his assistant, with two or three men-servants to clean the men's wards. It contains one hundred patients; of which, ten on an average are annually dismissed cured. It is the principle of the director never to contradict a patient, but to appear to obey and execute his most extravagant wishes. The greater part of the patients enter the hospital with the strongest antipathies against some friend or public person; suspect a conspiracy against their lives or fortunes; and urge or plan the death or ruin of the persons exciting their resentment. The director patiently listens to their complaints, offers to execute their orders, and thus quickly gains an ascendancy over them. When their dress is worn out, he renews it in form and colour precisely as they entered the hospital. They are allowed food at any hour they think proper, by night as well as by day. Plenty of water is always placed in their rooms. One of the most difficult things is to induce them, at first, to keep their rooms clean; they are apt to do every thing where it should not be done. He gets the better of them by this easy management. There is a lad, nearly an idiot, that goes about the house and digs in the garden: when a room is dirty, immediately the director tells a patient, he is very sorry that the poor idiot has contrived to slip into the room and dirty it; he entreats the patient to watch well that the idiot does not return; he scolds the boy, and this, repeated two or three times, almost always induces the patient to be cleanly. Neither straight-waistcoats, nor ropes, nor chains, are ever used. There is a long gallery, with a range of small rooms on one side, and of larger rooms opposite. An outrageous patient is merely confined in a small room, in which is a bedstead, chair, and table, all screwed to the floor, with a straw mattress and blankets; no glass is in the windows, but iron bars, and outside Venetian blinds that can be closed so as nearly to exclude the cold air, if necessary. When the patient is quiet, he is allowed to cross the gallery into the opposite room, while his own is cleaning out; when convalescent, the patients walk in an open gallery,—in good weather, in a garden, and attend regularly in the chapel of the hospital. The great object in view is to keep the mind of the patient free from irritation, by giving him food whenever he chooses, and by appearing to obey his wishes. They always urge, that his bodily health requires his remaining in their house. They never beat or threaten a patient, but impute, before him, any misbehaviour of his to another person. Five of the sisterhood attend daily by rotation. Meat and soups are kept warm in the kitchen night and day. Weak wine is allowed in moderation. Little or no medicine is used beyond common purgatives. A physician calls daily, but is not exclusively attached to the establishment.

---

