# Errata

**Published:** 1818-12

**Authors:** 


					302, ? 28, president, add, of a society composed of the
members of.'
322, ? 35, for IF. read A. C.

				

